# Rectal-sparing type of ulcerative colitis predicts lack of response to pharmacotherapies

**DOI:** 10.1186/s12893-017-0255-5

**Published:** 2017-05-19

**Authors:** Yuki Horio, Motoi Uchino, Toshihiro Bando, Teruhiro Chohno, Hirofumi Sasaki, Akihiro Hirata, Yoshio Takesue, Hiroki Ikeuchi

**Affiliations:** 1 Department of Inflammatory Bowel Disease, Hyogo College of Medicine, 1-1, Mukogawa-cho, Nishinomiya, Hyogo 663-8501 Japan; 2 Infection Control and Prevention, Hyogo College of Medicine, Hyogo, Japan

**Keywords:** Ulcerative colitis, Rectal-sparing, Urgent/emergent surgery, Inflammatory bowel disease

## Abstract

**Background:**

Ulcerative colitis (UC) is known as an immune disorder of the colon that generally involves the rectum, but an atypical distribution of inflamed mucosa has previously been noted in certain subtypes of UC, such as the rectal-sparing type (RST). As noted in a previous report, patients with the RST may be at elevated risk for disease refractoriness, but the clinical significance of RST remains unknown.

**Methods:**

UC patients who underwent surgery between January 2010 and April 2015 were included. Patients were classified as having the RST or a non-RST based on colectomy specimens or a pre-operative endoscopy. Possible risk factors for urgent/emergent surgery were analyzed. We specifically determined whether the RST is a significant predictor for urgent/emergent surgery.

**Results:**

In total, 46/482 patients were classified as having the RST. Disease severity was significantly worse in patients with the RST than in other patients (*p = 0.02*). Urgent/emergent surgery was required for 24/46 patients with the RST, compared with 107/436 non-RST patients (*p < 0.01*). The overall incidence of urgent/emergent surgery was 131/482. Disease duration < 70.2 months [odds ratio (OR) 2.45], severe disease (OR 87.1), total administered steroid dose < 5000 mg (OR 3.02), daily pre-operative steroid dose ≥ 9 mg (OR 2.59), and the RST (OR 5.59) were identified as independent risk factors for urgent/emergent surgery.

**Conclusion:**

The RST was an independent risk factor for urgent/emergent surgery in our analysis of surgically treated patients with UC.

## Background

Ulcerative colitis (UC) is known as an immune disorder of the colon that generally involves the rectum. UC is typically classified into several categories based on the extension of colitis, which includes proctitis, including left-side colitis, pan-colitis, or segmental colitis [1]. Generally, inflammation arising from distal proctitis can diffusely or continuously extend to the proximal colon. However, an atypical distribution of inflamed mucosa has been noted previously in various conditions, such as the rectal-sparing type (RST), even in the absence of topical treatment for proctitis [2]. In a previous report, although RST could be recognized in endoscopic examinations, histological inflammation always existed in biopsy specimens [3]. Therefore, the RST may be indicated by relatively minor inflammatory findings in an endoscopic examination.

Previous reports have suggested an association between primary sclerosing cholangitis (PSC) and the RST [4–6]. PSC exhibits a high risk of colitis-associated cancer [7–9] and pouchitis after a restorative proctocolectomy [10]. Although remission of rectal inflammation can be achieved via topical treatments, including aminosalicylate and corticosteroids, the RST may be observed at the time of disease onset, even in the absence of any treatments; the condition is also more frequently noted in pediatric patients [11–14]. Although RST patients exhibited a high risk for refractory disease in a previous report [15], the clinical significance of the RST remains unknown.

We recently treated several UC patients with the RST who required urgent/emergent surgery. Therefore, we retrospectively reviewed clinical records to evaluate clinical features of RST patients and the association between the RST and failure of medical therapy (i.e., need for colectomy).

## Methods

### Patients

UC patients who underwent a laparotomy at Hyogo College of Medicine between January 2010 and April 2015 were included in this study. All colectomy specimens with histologic features of UC and no Crohn’s-like features such as granulomas, transmural lymphoid aggregates, or fissures were identified. Gender, age at onset of UC, age at initial surgery, duration of disease, disease severity, total administered prednisolone (PSL) dose, daily pre-operative PSL dose, immunomodulator administration, biologic administration, and timing of surgery, including whether surgery was urgent/emergent or elective, were retrospectively determined using clinical records. UC disease activity was assessed primarily according to clinical features using the criteria of Truelove and Witts [16]. Total administered corticosteroids were converted into the PSL dose and calculated based on the administered corticosteroid dose since the initial onset of UC. The types of immunomodulators, including calcineurin inhibitors and thioprines, and biologics administered were determined based on medication history, irrespective of the dosing period.

### Definition of the RST of UC

Patients were classified as having the RST or a non-RST (i.e., ordinary diffuse proctocolitis). The RST was noted when the rectum was obviously spared of active or chronic inflammation, and relatively little or no inflammation was observed at the rectum compared to proximal colitis in colectomy specimens. Alternatively, the RST was noted when rectal inflammation was limited to a Mayo endoscopic score of 0 or 1 and was also milder than that of the proximal colon based on pre-operative endoscopy. All endoscopic findings were assessed by expert physicians at our institution who were familiar with inflammatory bowel disease. The RST was exclusively characterized by unmagnified visual findings regardless of histological findings.

### Primary outcome

The primary end point was urgent/emergent surgery. Possible risk factors for urgent/emergent surgery, including gender, age at onset of UC, age at initial surgery, duration of disease, disease severity, existence of the RST, total administered PSL dose, daily pre-operative PSL dose, and immunomodulator or biologic administration, were analyzed to identify significant predictors. Tacrolimus (TAC) or cyclosporine (CsA) is administered as rescue therapy for severe steroid-refractory UC, and azathioprine (AZA) or 6-mercaptopurine (6-MP) is used to maintain remission. Therefore, we analyzed immunomodulator administration based on two categories: TAC/CsA and AZA/6-MP.

### Surgical indications and timing of surgery

At our institution, the absolute indications for emergent surgery include massive hemorrhage and free perforation. Indications for urgent surgery performed within 24 h of admission include severe or fulminant colitis with or without toxic megacolon that is unresponsive to conventional maximal pharmacotherapies and less severe colitis with medically intractable or intolerable pharmacotherapy side effects with a progressively worsening general condition. Elective surgery was indicated for refractory disease or extra-intestinal manifestations without a progressively worsening general condition or complicated carcinoma or dysplasia. The standard surgical procedures for UC at our institution include total proctocolectomy and ileal pouch anal anastomosis (IPAA) with or without an ileostomy. A total colectomy with mucous fistula and end ileostomy was mainly performed as an emergency surgery. This procedure was performed for patients with free perforation, toxic megacolon, or fulminant colitis with progressive worsening to severe sepsis or septic shock.

### Exclusion criteria

We excluded patients from the RST group who were treated with topical treatments.

All colectomy specimens were identified as UC based on histological features. Patients with a diagnosis or suspicion of Crohn’s disease based on histological findings were not included in this series.

### Statistical analysis

The statistical analysis was performed as follows. Categorical variables were compared using a chi-squared test. Numerical variables are expressed as medians and ranges and were compared using the Mann-Whitney U test. The cut-off values for age at onset of UC, age at initial surgery, duration of disease, total administered PSL dose, and pre-operative PSL dose were defined as the median values in this series (32 years, 44 years, 70.2 months, 5000 mg, and 9 mg, respectively). The level of statistical significance was set at *p < 0.05*. The odds ratio (OR) and 95% confidence intervals (CIs) were calculated for all variables in the univariate analysis. Each individual factor with a significant *p-value* in the univariate analyses was subsequently entered into a stepwise logistic regression model. JMP ver.12 (SAS Institute Inc., Cary, North Carolina, USA) was used to perform all analyses.

### Ethical statement

All study protocols were approved by the institutional review board of Hyogo College of Medicine (no. 2078). Informed consent and approval for the use of patient data were obtained prior to surgery.

## Results

### RST characteristics

**Fig. 1:**
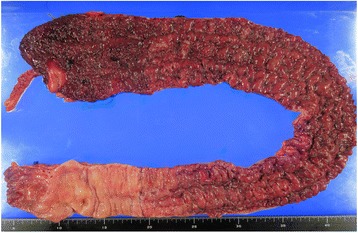
A typical case of the rectal-sparing type of ulcerative colitis: the rectum was clearly spared from active or chronic inflammation, with relatively little to no inflammation of the rectum compared with that observed for the proximal colon in colectomy specimens

A total of 482 patients who underwent a colectomy were analyzed. The clinical characteristics of patients are presented in Table 1. Overall, 46/482 (9.5%) patients had the RST of UC. Figure 1 presents a typical RST colectomy specimen. No significant differences in gender, age at onset of UC, and age at initial surgery were noted between the RST and non-RST groups. The time from onset of UC to initial surgery was significantly shorter for patients with the RST than for patients with a non-RST (*p < 0.01*). The disease severity was classified as severe in 18 patients (39.1%) and fulminant 3 (6.5%) patients with the RST, resulting in a significantly higher rate of severe disease in RST patients (*p = 0.02*).

**Table 1:** Patients characteristics

	All patients *n* = 482	RST *n* = 46	non RST *n* = 436	*p- value*
Gender (Male/Female)	316/166	29/17	287/149	*0.74*
Age at onset of UC (years)	35.7 ± 17.4	35.7 ± 18.8	35.7 ± 17.3	*0.99*
Age at initial surgery (years)	44.6 ± 16.7	41.2 ± 19.3	44.9 ± 16.4	*0.15*
Disease duration (months)	70.2 (0.2-479.7)	31.4 (1.1-347.3)	73.6 (0.2-479.7)	<0.01*
Severe/Fulminant disease	145(30.1)	21(45.7)	124(28.4)	0.02*
Total givenPSL dose (mg)	5000 (0-200,000)	4535 (300-47,000)	5000 (0-200,000)	*0.76*
Daily pre-opearative PSL dose (mg/day)	9(0-80)	12.5(0-80)	7.5(0-80)	*0.38*
Immunomodulators administration	247(51.2)	30(65.2)	217(49.8)	0.04*
Biologics administration	127(26.3)	11(23.9)	116(26.6)	*0.69*
Surgical indication				
cancer/dysplasia	110(22.8)	2(4.3)	108(24.8)	<0.01*
free perforation	27(5.6)	2(4.3)	25(5.7)	
refractory disease	343(71.2)	42(91.3)	301(69.0)	
EIMs	2(0.4)	0	2(0.4)	
Urgent/emergent surgery	131(27.2)	24(52.2)	107(24.5)	<0.01*

RST = rectal sparing type, UC = ulcerative colitis, PSL = prednisolone, EIMs = extra intestinal manifestations

Data are numbers with percentages in parentheses unless otherwise indicated

Continuous variables are indicated as mean ± standard deviation and median (range)

* *p < 0.05* (Significantly different)

Regarding pharmacotherapy, patients with the RST were significantly more likely to have received immunomodulators (*p = 0.04*), but the other therapies were not significantly different between the groups. The surgical indication was cancer in 1 (2.1%) patient and dysplasia in 1 (2.1%) patient with the RST. In contrast, among subjects with a non-RST, the surgical indication was cancer in 64 (14.7%) patients and dysplasia in 44 (10.1%) patients. In addition, urgent/emergent surgery was required for 24/46 (52.2%) patients with the RST and 107/436 (24.5%) patients with a non-RST; the rate of urgent/emergent surgery was significantly higher among patients with the RST (*p < 0.01*).

### Primary outcome

The overall incidence of urgent/emergent surgery was 131/482 (27.2%). Univariate and multivariate analyses were performed to identify independent risk factors for urgent/emergent surgery. The results of univariate analyses of risk factors potentially associated with urgent/emergent surgery are presented in Table 2. Age at initial surgery < 44 years, duration of disease < 70.2 months, severe/fulminant disease, total administered PSL dose < 5000 mg, daily pre-operative PSL dose ≥ 9 mg/day, and the RST were significant risk factors for urgent/emergent surgery. Patients who underwent elective surgery were significantly more likely to have received AZA/6-MP *(p < 0.01)*. TAC/CsA administration was not significantly different between the groups. In addition, the seven statistically significant factors mentioned above were entered into the multivariate logistic regression analysis (Table 3). Duration of disease < 70.2 months (OR = 2.45, 95% CI 1.10-5.54, *p = 0.03*), severe/fulminant disease (OR = 87.1, 95% CI 40.9-205.1, *p < 0.01*), total administered PSL dose < 5000 mg (OR = 3.02, 95% CI 1.39-6.76, *p < 0.01*), daily pre-operative PSL dose ≥ 9 mg (OR = 2.59, 95% CI 1.20-5.65, *p = 0.02*), and the RST (OR 5.59, 95% CI 1.90-16.9, *p < 0.01*) were identified as independent risk factors for urgent/emergent surgery.

**Table 2:** Univariate analysis for risk factors associated with Emergent/Urgent surgery

Risk factors	urgent/emergent surgery *n* = 131	elective surgery *n* = 351	*p- value*	OR (95%CI)
Gender male	88(67.1)	228(64.9)	*0.64*	1.10 (0.72-1.69)
Age at initial surgery < 44 years	80(61.1)	158(45.0)	<0.01*	1.92 (1.28-2.89)
Age at onset of UC < 32 years	55(41.9)	181(51.6)	*0.06*	0.68 (0.45-1.02)
Duration of disease < 70 months	105(80.2)	136(38.7)	<0.01*	6.38 (3.95-10.32)
Severe/Fulminant disease	118(90.1)	27(7.7)	<0.01*	108.9 (56.3-2227.1)
Rectal sparing type	24(18.3)	22(6.3)	<0.01*	3.35 (1.81-6.26)
Total given PSL dose <5000 mg	91(69.5)	132(37.6)	<0.01*	3.77 (2.46-5.80)
Daily pre-operative PSL dose ≥ 9 mg/day	103(78.6)	139(39.6)	<0.01*	5.61 (3.55-9.11)
Immunomodulators				
TAC/CsA	39(29.8)	84(23.9)	*0.19*	1.35 (0.86-2.09)
AZA/6-MP	16(12.2)	108(30.8)	<0.01*	0.32 (0.17-0.54)
Biologics administration	33(25.2)	194(55.3)	*0.72*	1.08 (0.68-1.72)

OR = Odds Ratio; CI = Confidence Interval; PSL = Prednisolone; TAC = Tacrolimus; CsA = Cyclosporine; AZA = Azathioprine 6-MP = 6-Mercaptopurine

Data are numbers with percentages in parentheses unless otherwise indicated

*p < 0.05 (Significantly different)

**Table 3:** Multivariate analysis for risk factors associated with urgent/emergent surgery

Risk Factors	OR(95% CI)	*p-value*
Age at initial surgery <44 years	1.94(0.93-4.17)	*0.08*
Duration of disease(month) < 70 months	2.45(1.10-5.54)	0.03*
Severe/Fuluminant disease	87.1(40.9-205.1)	<0.01*
Total given PSL dose <5000 mg	3.02(1.39-6.76)	<0.01*
Daily pre-operative PSL dose ≥9 mg/day	2.59(1.20-5.65)	0.02*
Immunomodulators administration (AZA/6-MP)	0.68(0.26-1.69)	*0.41*
Rectal sparing type	5.59(1.90-16.9)	<0.01*

OR = Odds Ratio; CI = Confidence Interval; PSL = Prednisolone; AZA = Azathioprine;6-MP = 6-Mercaptopurine

*p < 0.05 (Significantly different)

## Discussion

The RST of UC may occur at some point during the medical treatment of UC in as many as 44% of cases [17]. This result may indicate that only the timing of the disease or the extent of disease may be revealed by treatment modifications. According to a previous report, the RST exclusively exhibits one course of disease progression because histological inflammation was consistently defined. These results suggest that an absolute RST does not exist, and most RST cases consist of a relative RST [18, 19]. In addition, Park et al. [18]. reported that most RST patients develop remarkable, visible inflammation during disease progression. Therefore, we first excluded patients who received topical treatments from the RST group. Unfortunately, the initial degree of rectal inflammation, alteration and progression of the disease, and timing of RST appearance before surgery could not be evaluated retrospectively. However, RST diagnosed immediately before surgery was an independent risk factor for urgent/emergent surgery in this series. Urgent/emergent surgery may indicate refractoriness and resistance to pharmacotherapies. Oshitani et al. [15] reported that patients with endoscopically defined RST exhibited enhanced refractoriness and increased recurrence rates and had a longer duration of active disease than patients without the RST. Therefore, presence of the RST may predict a future colectomy.

Given the effects of several immunosuppressive therapies, associations between such therapies and the RST should be discussed. Almost all of the included patients received immunosuppressive treatment prior to surgery; however, their medical therapy failed, resulting in colectomy. It is difficult to envision that immunosuppressive treatment, with the exception of enema therapy, was effective in only the rectal segment. If immunosuppressive treatment had been effective, patients would have exhibited improved symptoms and would not have required surgery, and endoscopy would also have revealed remission in not only the rectum but also the entire colonic area.

Regarding the definition of the RST, we used surgical specimens or endoscopic findings from shortly before surgery to assess patients treated via colectomy alone. Although diagnosis of the RST was defined as relatively reduced or no inflammation of the rectum compared to proximal colitis, Joo and Odze [19] reported that all RST patients were classified as having a relative RST, which indicates the existence of histological inflammation, even to a mild degree. Additionally, those researchers observed no correlations between the endoscopic findings and the colectomy histological findings [19]. They concluded that absolute rectal sparing does not occur in UC, even after the long-term use of anti-inflammatory medications [19]. In this series, histological inflammation of the rectum was also defined in all RST patients; however, the inflammation was relatively mild compared to that of the proximal colon. We also conjecture that degrees of inflammation may be difficult to classify via histological findings alone or that most such findings may be classified as indicative of inflamed mucosa. Therefore, we considered the diagnosis and definition of RST as adequate, despite the limitations mentioned below.

Some limitations were present in this study. First, this was a retrospective study with small number of patients from a single center. Multicenter and prospective cohort studies are needed. Second, the indications for urgent surgery were slightly vague. The selection and duration of conservative treatments for refractory disease before surgery, the timing of urgent surgery, or the decision to perform surgery may differ among patients, institutions, physicians, or surgeons. Third, although we believe that our definition was adequate, the definition of the RST was slightly vague. The distinction was often difficult and might be inappropriate in patients with boundary findings between 0 and 1 for the Mayo endoscopic score. The distinction of resected surgical specimens might also be inappropriate, given that the conditions were defined based on the subjective impression of surgeons and histologists.

Our results suggest that a shorter duration of colitis, a low dose of total PSL, a high dose of pre-operative PSL, severe disease, and the RST are risk factors for urgent/emergent surgery. These results may reveal a lack of response to pharmacotherapies for treatment of the RST, or these patients may develop more extensive and rapid unresponsiveness to pharmacotherapies. Therefore, we should be aware that patients with an initial onset of UC whose colitis exhibits the RST and worsens within a short period may have a higher risk for further surgical intervention.

## Conclusion

The RST of UC was an independent risk factor for urgent/emergent surgery based on an analysis of surgically treated UC patients. Although the developmental mechanism and clinical significance of the RST remain unclear, these findings may suggest the need for a future colectomy, disease refractoriness, and pharmacotherapy resistance.

## Abbreviations

UC: Ulcerative colitis
RST: Rectal-sparing type
PSL: Prednisolone
PSC: Primary sclerosing cholangitis
EIMs: Extra-intestinal manifestations
TAC: Tacrolimus
CsA: Cyclosporine
AZA: Azathioprine
6-MP: 6-Mercaptopurine
IPAA: Ileal pouch anal anastomosis
OR: Odds ratio
CI: Confidence interval
